# NIR‐Excited Imaging of Cervical Cancer Cells Using Biocompatible LaF_3_:Er^3+^,Yb^3+^ Upconversion Nanophosphors

**DOI:** 10.1002/bio.70269

**Published:** 2025-07-25

**Authors:** Pratik Deshmukh, Bhumika Sharma, Sourabrata Chakraborty, Himanshu Srivastava, Khageswar Sahu, Srinibas Satapathy, Shovan Kumar Majumder

**Affiliations:** ^1^ Laser Biomedical Applications Division Raja Ramanna Centre for Advanced Technology Indore Madhya Pradesh India; ^2^ Training School Complex, Anushakti Nagar Homi Bhabha National Institute Mumbai Maharashtra India; ^3^ Accelerator Physics and Synchrotrons Utilization Division, Raja Ramanna Centre for Advanced Technology Indore Madhya Pradesh India

**Keywords:** bioimaging, cellular uptake, erbium (Er^3+^), HeLa cells, LaF_3_, upconversion nanoparticles (UCNPs), ytterbium (Yb^3+^)

## Abstract

Bioimaging plays a vital role in understanding complex biological processes, from tracking cellular activity to studying entire organisms. To improve the sensitivity and specificity of imaging techniques, researchers are increasingly turning to nanomaterials as contrast agents. Among them, upconversion nanoparticles (UCNPs) stand out for their ability to convert near‐infrared (NIR) light into visible emission. This property enables deep tissue penetration, reduced autofluorescence, and low phototoxicity. We report the synthesis of LaF_3_ codoped with 2 mol% Er^3+^ and 20 mol% Yb^3+^ using a hydrothermal method, followed by surface modification with human serum albumin (HSA). Structural analysis confirms the formation of rhombohedral LaF_3_ with uniform dopant incorporation, and electron microscopy reveals a near‐spherical morphology with average particle sizes increasing from ~25 nm (bare) to ~60 nm (HSA‐coated). Under 976‐nm excitation, the nanophosphors exhibit bright green and red UC emissions via a two‐photon mechanism. Cytotoxicity studies demonstrate high cell viability at concentrations up to 0.4 mg/mL in HeLa cells, while fluorescence uptake assays indicate time‐dependent internalization, peaking at 6‐h post‐treatment. These findings highlight the potential of HSA‐coated LaF_3_:Er^3+^,Yb^3+^ nanophosphors as efficient, biocompatible contrast agents for NIR‐excited luminescent bioimaging.

## Graphical abstract

This study demonstrates the potential of LaF_3_:2%Er^3+^,20%Yb^3+^ upconverting nanophosphors (UCNPs) as biocompatible contrast agents for bioimaging. Hydrothermally synthesized and human serum albumin (HSA)‐coated, the UCNPs exhibit strong upconversion luminescence, good biocompatibility, and efficient cellular uptake, making them promising candidates for further biomedical applications.

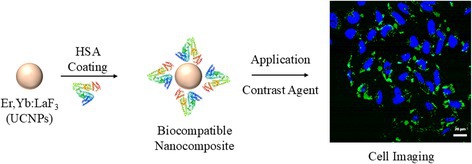

## 1 Introduction

The field of bioimaging is crucial in the visualization of biological activities, ranging from cellular interactions to the observation of whole organisms. This domain utilizes a variety of imaging modalities, such as magnetic resonance imaging (MRI), computed tomography (CT), ultrasonography, positron emission tomography (PET) and optical imaging techniques [1, 2]. Nanoparticles are increasingly utilized as contrast agents to enhance the sensitivity and specificity of these imaging methods [3, 4, 5]. Among these, upconversion nanoparticles (UCNPs) are emerging as promising contrast agents for optical imaging due to their unique properties [6, 7, 8, 9]. UCNPs are luminescent materials with the remarkable ability to convert lower‐energy photons, typically in the near‐infrared (NIR) region, into higher‐energy photons like ultraviolet (UV) or visible light [6, 10]. This phenomenon, termed anti‐Stokes luminescence, makes them attractive because of their absorption within the “biological window” (900–1200 nm) [11]. This leads to several advantages, including reduced background interference, enhanced tissue penetration along with minimized phototoxicity and photobleaching [12].

UCNPs are typically composed of lanthanide ions doped into a nanosized crystalline inorganic host material (1–100 nm) [13]. The lanthanide ions possess unique energy level structures that enable the anti‐Stokes luminescence process. When excited with multiple low‐energy photons, these ions can absorb the energy sequentially and emit a single higher‐energy photon [13, 14]. The efficiency of this UC process is significantly influenced by the phonon energy of the host material. Lower phonon energy minimizes nonradiative energy transfer pathways, leading to brighter UC luminescence [15]. Thus, fluoride and oxide‐based materials are preferred hosts for NIR‐to‐visible UC due to their inherently lower phonon energies [16, 17, 18, 19].

Within the group of lanthanides, erbium ions (Er^3+^) have been extensively studied for their role as an active ion in phosphors due to their sequential energy levels. When excited by 970 nm, Er^3+^ ions exhibit emissions in the green (^2^H_11/2_, ^4^S_3/2_ → ^4^I_15/2_) and red (^4^F_9/2_ → ^4^I_15/2_) regions of the visible spectrum [20]. However, the relatively low absorption cross‐section of Er^3+^ limits its practical applicability [20, 21].

A common strategy to enhance the absorption cross‐section involves increasing the concentration of lanthanide ions in the host material. However, this approach can lead to concentration quenching, where excess ions cause a decrease in the overall fluorescence output [22, 23]. This quenching occurs due to nonradiative energy transfer between lanthanide ions, leading to a loss of excitation energy. To overcome this limitation, researchers often employ a combination of lanthanide dopants. In this scenario, one ion, known as a sensitizer (e.g., Yb^3+^, Nd^3+^), efficiently absorbs incident NIR light and transfers the energy to another ion, termed the activator (e.g., Er^3+^, Ho^3+^, Tb^3+^), which is responsible for the final emission [24, 25]. Yb^3+^ (sensitizer) enhances Er^3+^ (activator) UC efficiency due to its larger absorption cross‐section at 980 nm [25]. The 20 mol% Yb^3+^ concentration aligns with widely reported optima for fluoride hosts, where this level maximizes energy transfer to Er^3+^ while avoiding concentration quenching from excessive Yb^3+^ –Yb^3+^ energy migration [26]. In LaF_3_, doping up to 21 mol% Yb^3+^ preserves the hexagonal crystal structure, as confirmed by XRD analysis, ensuring minimal lattice distortion and efficient energy transfer pathways [27]. On the other hand, Er^3+^ concentrations above ~2 mol% often lead to cross‐relaxation losses, which suppress green emission and favor red emission [28]. The 2 mol% Er^3+^ minimizes this effect while maintaining sufficient activator density. Further, the optimized dopant concentrations for activator and sensitizer in commonly reported host material such as NaYF_4_, NaGdF_4_, BiF_3_, and K_3_ZrF_7_ are typically ~2 mol% and 20 mol%, respectively [6, 10, 13, 16, 20]. Accordingly, 2 mol% Er^3+^ and 20 mol% Yb^3+^ were chosen for codoping in LaF_3_.

In this study, Er^3+^ and Yb^3+^ codoped LaF_3_ nanophosphors were synthesized using a hydrothermal method, with a specific focus on their potential as contrast agents in bioimaging applications. Human serum albumin (HSA), the most abundant plasma protein, was employed for surface coating of the nanoparticles to enhance their biological compatibility. HSA is a 67 kDa, 585‐amino acid globular protein with approximate dimensions of ~8 × 8 × 3 nm [29, 30]. Its flexible structure, multiple binding sites, and net negative charge at physiological pH make it particularly well‐suited for nanoparticle surface functionalization, improving colloidal stability, biocompatibility, and enabling targeted delivery [29].

These HSA‐coated nanophosphors were subsequently evaluated for their intracellular uptake in human cervical cancer (HeLa) cells. The objective of this research is to assess the potential of these engineered nanophosphors to enhance contrast in bioimaging, thereby contributing to the advancement of biomedical imaging technologies.

## 2 Experimental

LaF_3_ nanoparticles doped with Er^3+^ and Yb^3+^ (LaF_3_:Er,Yb) were synthesized using a modified version of a previously reported method for LaF_3_:Tb/Yb nanoparticles [31]. These modifications aimed to achieve better control over nanoparticle size and agglomeration.

### 2.1 Materials

Following chemicals, as received from their respective sources, were utilized: lanthanum oxide (La_2_O_3_, Alfa Aesar, 99.99%), erbium oxide (Er_2_O_3_, Alfa Aesar, 99.99%), ytterbium oxide (Yb_2_O_3_, Alfa Aesar, 99.99%), ammonium fluoride (NH_4_F, Merck, 98%), ammonia solution (NH_3_, CDH, 25%), HSA (> 97%, MP Biomedicals), and nitric acid (HNO_3_, Merck, 69%).

### 2.2 Synthesis Procedure

Initially, a lanthanide precursor solution was prepared by dissolving Er_2_O_3_ (0.0301 g), Yb_2_O_3_ (0.3101 g), and La_2_O_3_(1 g) in dilute nitric acid (10 mL of 15 M acid in 50 mL of DI water). The specific stoichiometry was adjusted to achieve desired Er and Yb doping percentages (e.g., 2 mol% Er, 20 mol% Yb). Subsequently, a fluoride solution was prepared by dissolving NH_4_F (0.758 g) in 50 mL of DI water, followed by adjusting the pH to 8 using a few drops (~2 mL) of ammonia solution.

The lanthanide precursor solution was then added dropwise into the fluoride solution at a controlled rate of 5 mL/min while maintaining the temperature at 50°C. This process resulted in the formation of a milky white dispersion.

Following this, the total resulting mixture (~112 mL) was transferred to an autoclave and heated at 150°C for 1 h. After cooling to room temperature, the reaction mixture was washed three times with 100 mL of methanol per wash. The Er,Yb:LaF_3_ NPs were obtained by drying the washed product at 80°C for 5 h.

Finally, the coating of HSA onto the as‐synthesized NPs was carried out by the adsorption method as reported earlier with slight modifications [32]. Briefly, HSA was utilized to form a protein coating over the synthesized NPs. An HSA solution (5 mg/mL) in DI water was incubated with NPs (3 mg/mL) under constant stirring (250 rpm) at room temperature for approximately 12 h. The resulting HSA@Er,Yb:LaF_3_ nanocomposites were separated by centrifugation at 15,000 rpm for 5 min.

### 2.3 Characterizations

The crystal structure of the synthesized sample was analyzed using X‐ray diffraction (XRD) on a Rigaku diffractometer with Cu Kα radiation (*λ* = 1.5406 Å). Particle size distribution and zeta potential in aqueous medium were determined using a Malvern Zetasizer Nano ZS90 nanoparticle analyzer. A Carl Zeiss Sigma instrument for field emission scanning electron microscopy (FE‐SEM) was employed to examine particle size and morphology. The coating of HSA onto NPs was analyzed through morphological observations using transmission electron microscopy (TEM) performed at an acceleration voltage of 200 keV (Philips CM200). Elemental analysis was carried out using energy‐dispersive X‐ray spectroscopy (EDX) on the same FE‐SEM instrument. FTIR was carried out on Bruker ALPHA II spectrometer. Photoluminescence (PL) characterization involved a measurement of emission spectra under excitation by a 976‐nm continuous wave (CW) laser diode (1.5 W/cm^2^, spot diameter = 2 mm). The PL measurements were performed using a dispersion (0.3 mg/mL) of NPs in DI water, with an FLS920‐s fluorescence spectrometer (Edinburgh Instruments Ltd.).

#### 2.3.1 Cytotoxicity Evaluation

The cytotoxicity of the synthesized bare and HSA‐coated LaF_3_:Er^3+^,Yb^3+^ UCNPs was evaluated using the MTT (3‐(4,5‐dimethylthiazol‐2‐yl)‐2,5‐diphenyltetrazolium bromide) assay on HeLa cells. Approximately 5000 cells per well were seeded in a 96‐well plate and incubated for 24 h at 37°C with 5% CO_2_. Following incubation, the cells were treated with varying concentrations of both types of UCNPs (0.1, 0.2, 0.3, 0.4 and 0.5 mg/mL) prepared in DMEM (Dulbecco's Modified Eagle's Medium) for another 24 h under the same environmental conditions. After treatment, the MTT (0.5 mg/mL) was added to each well and the cells were incubated for an additional 6 h. The insoluble formazan salts formed by viable cells were then dissolved in dimethyl sulfoxide (DMSO). The absorbance of the formazan solution was measured at 590 nm using a microplate reader (Synergy HTX, BioTek). Lower absorbance values indicate lesser formazan production and, consequently, a lesser percentage of viable cells.

#### 2.3.2 Cellular Uptake Analysis

The cellular uptake of HSA‐coated LaF_3_:Er^3+^,Yb^3+^ UCNPs by HeLa cells was quantified using fluorescence spectroscopy. Briefly, HeLa cells were seeded at a density of 5000 cells per well (0.1 mL) in a 96‐well plate and allowed to adhere for 24 h. The culture medium was then replaced with fresh medium containing UCNPs dispersed in culture media at a concentration of 0.3 mg/mL. Cells were incubated with the UCNPs for varying incubation times at 37°C in a CO_2_ incubator. After incubation, the medium was removed, and the cells were washed three times with cold phosphate‐buffered saline (PBS) to remove unbound UCNPs. Subsequently, 0.2% Tween 20 lysis buffer was added to lyse the cells and release the internalized UCNPs. The fluorescence emission spectrum of the internalized UCNPs was measured using a fluorescence spectrometer (Edinburgh Instruments Ltd) upon excitation with a 976‐nm laser diode (1.5 W/cm^2^, spot diameter = 2 mm).

To further confirm cellular uptake, a confocal laser scanning microscope (Zeiss LSM 880) was utilized. HeLa cells were incubated with 0.3 mg/mL HSA‐coated Er,Yb:LaF_3_ NPs for 6 h and counterstained with Hoechst 33342 nuclear stain. A 405‐nm laser diode (power density: 5 mW/cm^2^ at sample plane) was used to excite Hoechst (emission band 410–500 nm), while a 488‐nm Ar laser (power density: 5 mW/cm^2^ at sample plane) was used to probe Er^3+^ in Er, Yb:LaF_3_ NPs (emission band: 530–560 nm).

#### 2.3.3 Statistical Analysis

Data were analyzed using Graph Pad (Version 9.0). All the data in histogram plots were expressed as mean and standard deviation. One‐way ANOVA analysis was conducted to determine statistical significance of the observed differences among different means. *, **, and *** denote *p* < 0.05, *p* < 0.01, and *p* < 0.001, respectively.

## 3 Results and Disscussion

### 3.1 XRD and EDX Analysis

**FIGURE 1:**
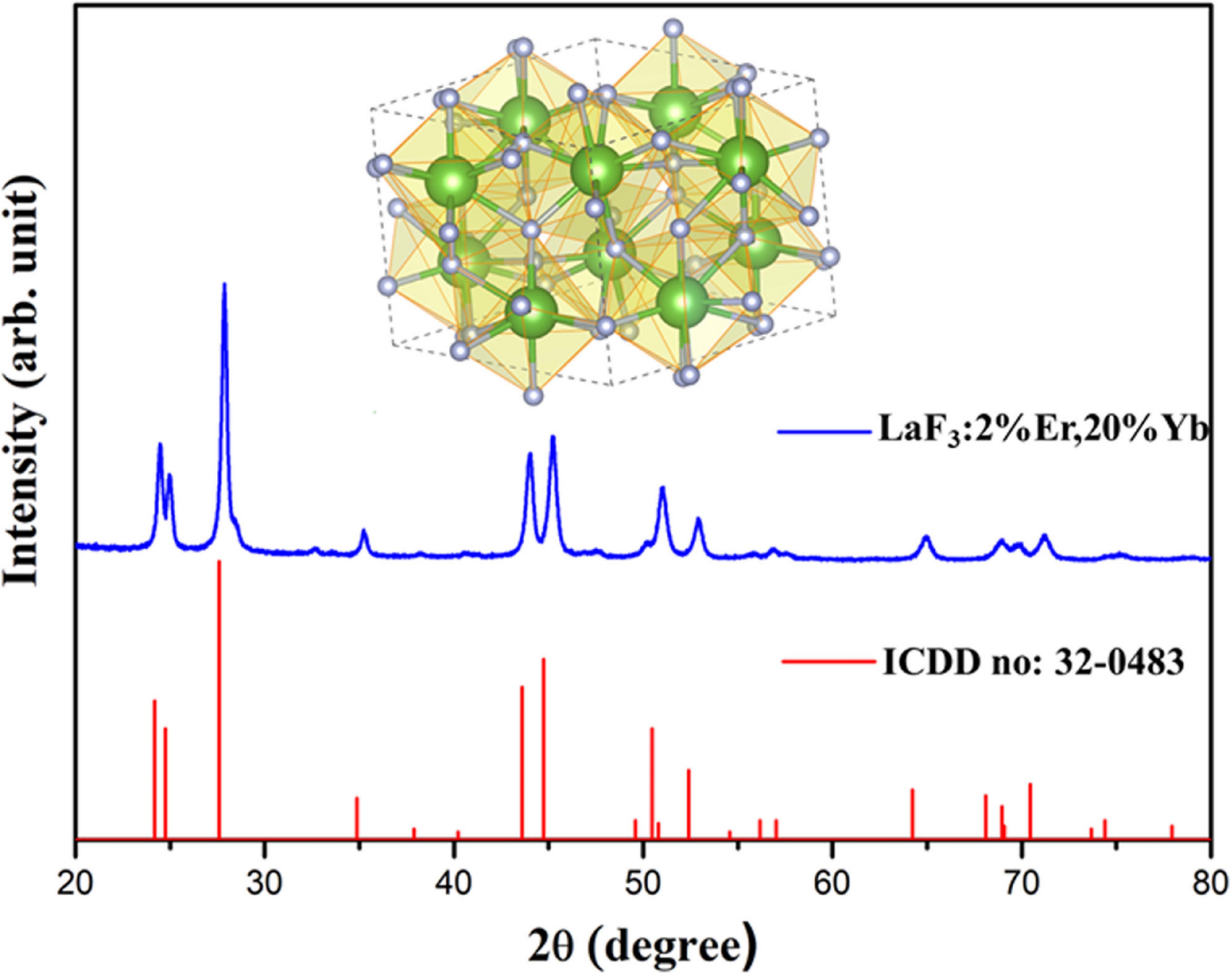
XRD spectrum of Er,Yb:LaF_3_; inset shows the crystal structure of LaF_3_.

The XRD pattern of the synthesized LaF_3_ sample codoped with 2 mol% Er^3+^ and 20 mol% Yb^3+^ (onwards will be termed as Er,Yb:LaF_3_) is presented in Figure 1.

**FIGURE 2:**
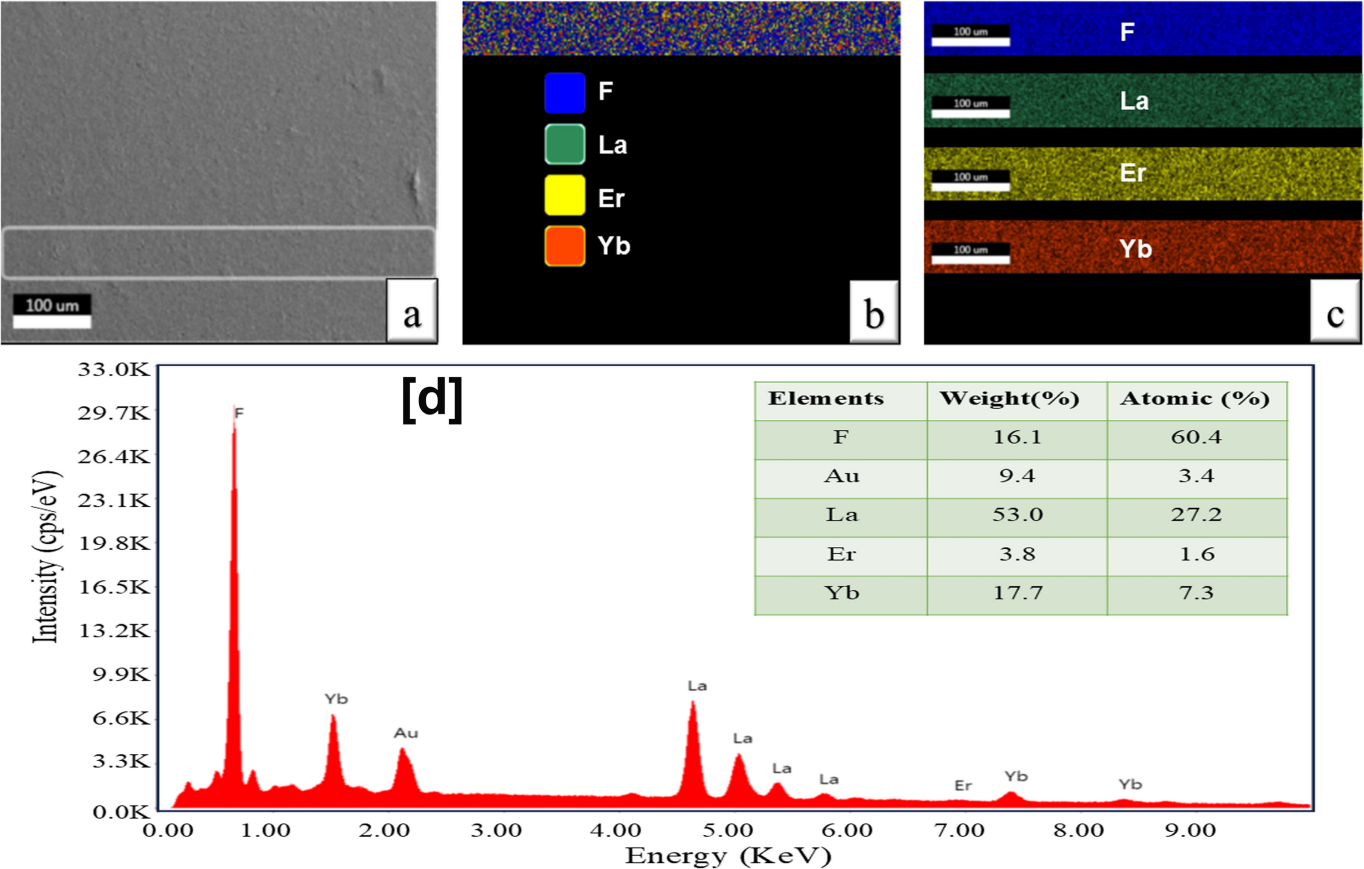
Elemental mapping of Er,Yb:LaF_3_ pellet: (a) FE‐SEM image of Er,Yb:LaF_3_ pellet. The inscribed rectangular region depicts the ROI for elemental mapping. (b) Mixed elemental mapping of ROI. (c) Individual mapping of fluorine (F), lanthanum (La), erbium (Er) and ytterbium (Yb) in Er,Yb:LaF_3_ pellet in the ROI. (d) EDX spectrum of Er,Yb:LaF_3_; inset shows relative elemental composition estimated from EDX spectrum.

The observed peaks and their positions closely match the reference pattern of ICDD File no. 32‐0483, indicating the formation of a rhombohedral phase LaF_3_ (space group *P* $\bar{3}$ *c*1) [33]. However, slight shifts in peak position are evident. This shift can be attributed to the variation in ionic radii between the dopant cations and La^3+^. Specifically, Yb^3+^ (116 pm, VI coordination) has a larger ionic radius compared to La^3+^ (103.2 pm, VI coordination), whereas Er^3+^ (100 pm, VI coordination) possesses a more comparable radius [34]. This observation suggests the successful substitution of La^3+^ by both Yb^3+^ and Er^3+^ within the LaF_3_ unit cell. Employing the Scherrer formula, the crystallite size estimated from the full width at half maximum (FWHM) of the (111) peak (2θ = 27.58°) is approximately 14 nm. Elemental mapping using EDX was conducted on a pellet (6 mm in diameter and 1 mm in thickness) prepared using Er,Yb:LaF_3_ NPs, as depicted in Figure 2a. The results illustrated a uniform distribution of elements such as lanthanum, erbium, ytterbium and fluorine, as shown in Figure 2b,c. The relative atomic percentages of these elements present in the nanophosphor were analyzed via the EDX spectrum, as shown in Figure 2d.

### 3.2 FE‐SEM, TEM, Particle Size Distribution, and Zetapotential Analysis

**FIGURE 3:**
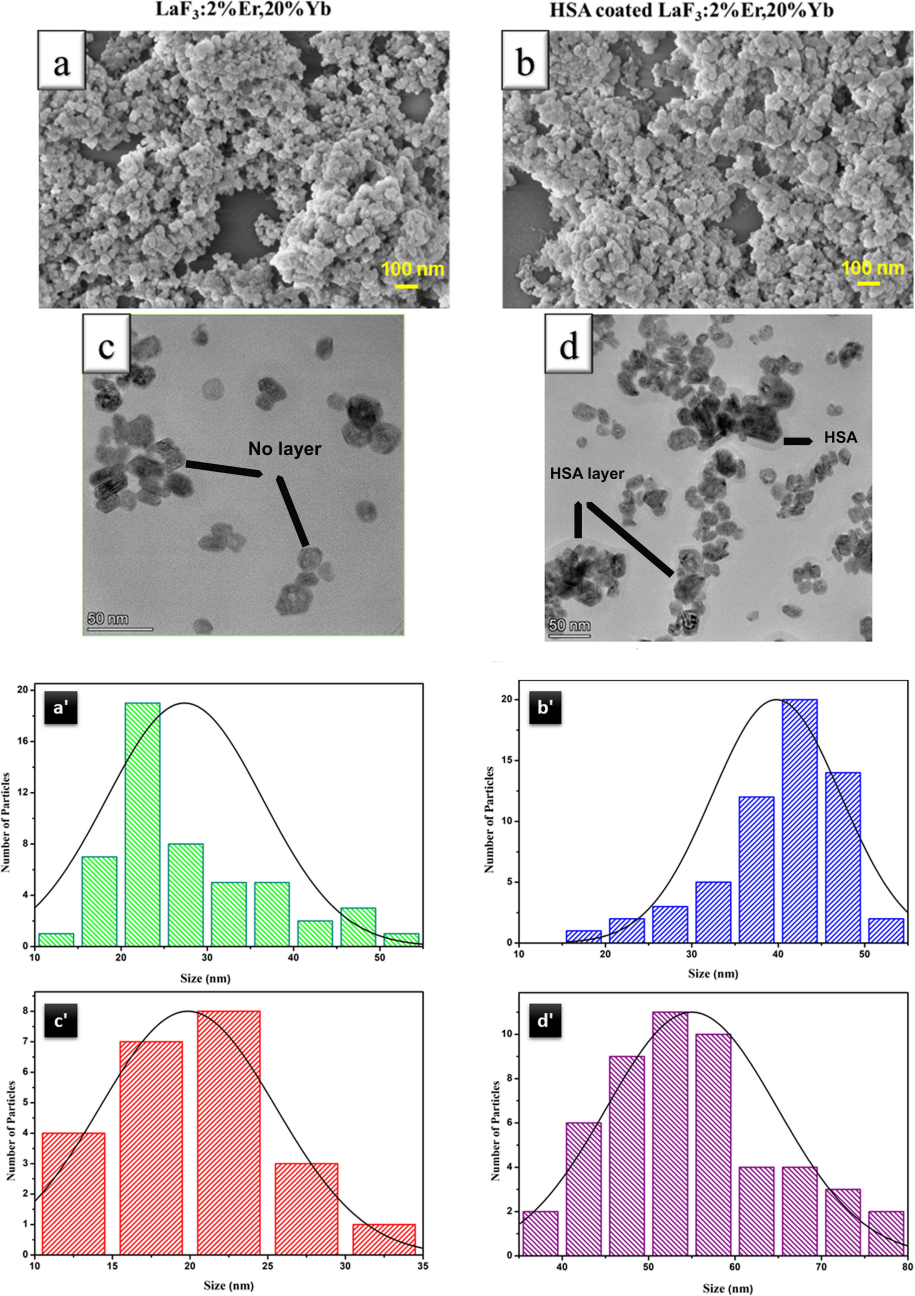
Morphological analysis and particle size histogram of bare and HSA‐coated Er,Yb:LaF_3_ NPs: (a) FE‐SEM image of bare Er,Yb:LaF_3_. (b) FE‐SEM image of HSA‐coated Er,Yb:LaF_3_ nanoparticles. (c) TEM image of bare Er,Yb:LaF_3_. (d) TEM image of HSA‐coated Er,Yb:LaF_3_ nanoparticles. Histrogram of particle size distribution derived from (a′) FE‐SEM image of bare Er,Yb:LaF_3_, (b′) FE‐SEM image of HSA‐coated Er,Yb:LaF_3_, (c′) TEM image of bare Er,Yb:LaF_3_, and (d′) TEM image of HSA‐coated Er,Yb:LaF_3_.

The particle size and morphology of synthesized sample were examined using FE‐SEM. A drop of aqueous solution of NPs (1 mg/mL prepared by dispersing in DI water) was placed onto a glass coverslip and allowed to dry. Finally, it was coated with a thin layer of gold using sputter deposition. In Figure 3a,b, both the bare, and HSA‐coated Er,Yb:LaF_3_ NPs displayed a near‐spherical form, with diameters approximately ranging from 20 to 30 nm and 30 to 100 nm, respectively. Their consistent size and shape distribution showcased the uniformity achieved through the hydrothermal synthesis technique. However, SEM images indicated some particle agglomeration, which is due to soft agglomeration of particles formed during sample preparation for FE‐SEM.

To examine the morphology of the core‐shell structure, TEM was utilized. TEM images of bare and HSA‐coated Er,Yb:LaF_3_ NPs are shown in Figure 3c,d. The bare NPs exhibit a narrow size distribution with an average diameter of ~20 nm. Upon HSA adsorption, the NPs aggregate due to the formation of HSA bridges. A uniform HSA coating layer, ~8 nm thick, is observed surrounding the NPs. Furthermore, size distribution histograms for both bare and HSA‐coated NPs were derived from SEM and TEM images using ImageJ software and are presented in Figure 3a′–d′.

For UNCPs to be an effective biological contrast agent, their size and stability in a biological environment are critical. Therefore, characterizing their particle size distribution and zeta potential is crucial.

For dynamic light scattering (DLS) and zeta potential measurements, dispersions of bare and HSA‐coated Er,Yb:LaF_3_ were prepared at a concentration of 1 mg/mL in DI water. This concentration was chosen to ensure accurate analysis of particle size distribution and zeta potential in a colloidal environment.

**FIGURE 4:**
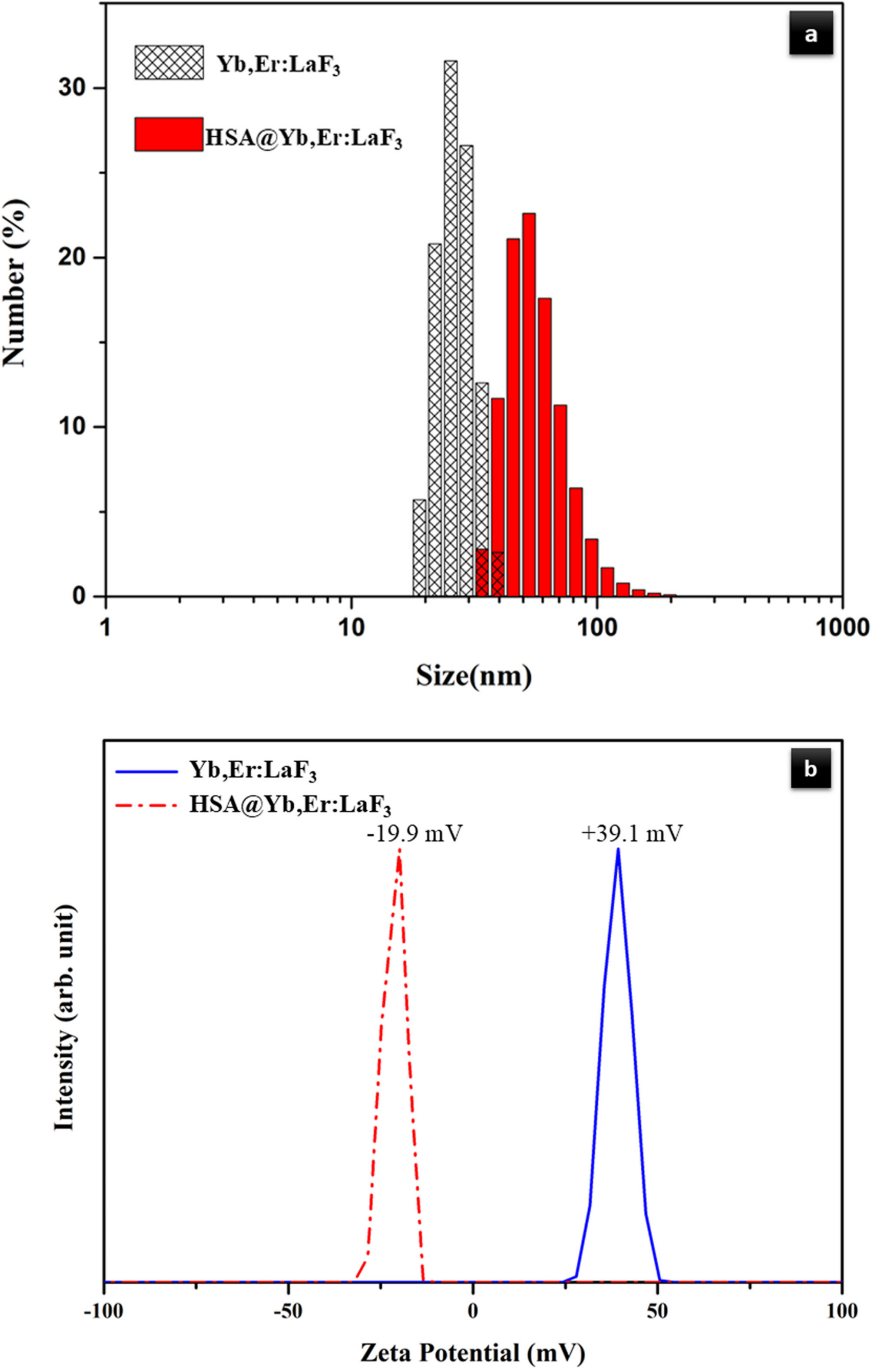
(a) Particle size distribution and (b) zeta potential plots of bare Er,Yb:LaF_3_ and HSA‐coated Er,Yb:LaF_3_ nanoparticles.

DLS analysis revealed a narrow and well‐defined size distribution of approximately 20–30 nm for bare Er,Yb:LaF_3_ NPs (Figure 4). This confirms the uniformity of the synthesized particles. Furthermore, DLS analysis verified the adsorption of HSA onto the NPs, as evidenced by the increase in size distribution after coating.

Zeta potential measurements demonstrated a positive value (+39.1 mV) for bare Er,Yb:LaF_3_ and a negative value (−19.9 mV) for HSA‐coated Er,Yb:LaF_3_ as shown in Figure 4b. The negative surface charge of the coated particles likely originates from the carboxylic acid (COO^−^) groups present in the HSA molecules [35, 36].

### 3.3 UV–Vis Absorbance and FTIR Analysis

**FIGURE 5:**
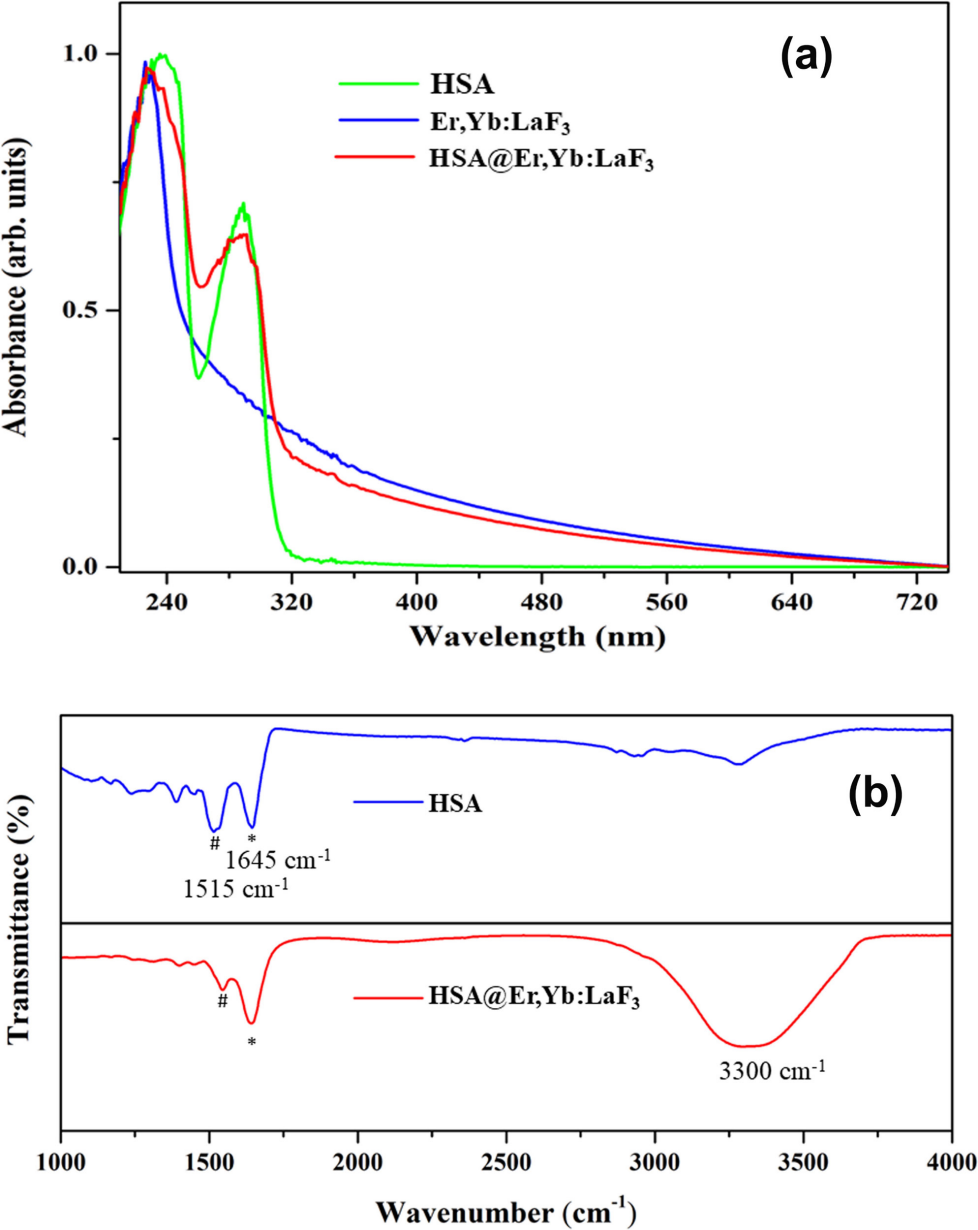
UV–Vis absorbance and FTIR: (a) UV–Vis absorption spectra of pure HSA, bare, and HSA‐coated Er,Yb:LaF_3_ nanoparticles. (b) FTIR spectra of pure HSA and HSA‐coated Er,Yb:LaF_3_ nanoparticles.

The normalized UV–vis absorption spectra of HSA, Er,Yb:LaF_3_, and HSA‐coated Er,Yb:LaF_3_ NPs dispersed in DI water (1 mg/mL) are displayed in Figure 5a. Albumin typically exhibits two primary absorption bands: one centered around 236 nm, corresponding to the *n* → π* transition of the C=O group, and another at 280 nm, attributed to the *π* → π* transition of aromatic amino acids such as tryptophan (Trp), tyrosine (Tyr), and phenylalanine (Phe). These characteristic absorption bands are distinctly observable in both the pure HSA sample and the HSA‐coated Er,Yb:LaF_3_ NPs. This observation confirms the successful coating of HSA onto the surface of Er,Yb:LaF_3_ NPs.

Further, FTIR was employed to characterize the functional groups and their bonding environments within the synthesized NPs. FTIR spectra were acquired for both pure HSA and HSA‐coated Er,Yb:LaF_3_ NPs (Figure 5b). A broad absorption band centered at approximately 3300 cm^−1^, observed in both spectra, is attributed to the O‐H stretching vibrations of adsorbed water molecules [37, 38]. The characteristic peaks for HSA are amide I and II bands, located around 1645 and 1515 cm^−1^ in Figure 5b. These bands arise due to protein backbone vibrations: amide I corresponds to C=O stretching coupled with N‐H bending, while amide II originates from N‐H bending and C‐N stretching [39, 40]. The presence of these amide I and II peaks in the FTIR spectrum of the HSA‐coated Er,Yb:LaF_3_ NPs (Figure 5b) confirms the successful attachment of HSA molecules onto the surface of the nanocrystals. This observation provides strong evidence for HSA ligand conjugation on the surface of UCNPs.

### 3.4 PL Analysis

**FIGURE 6:**
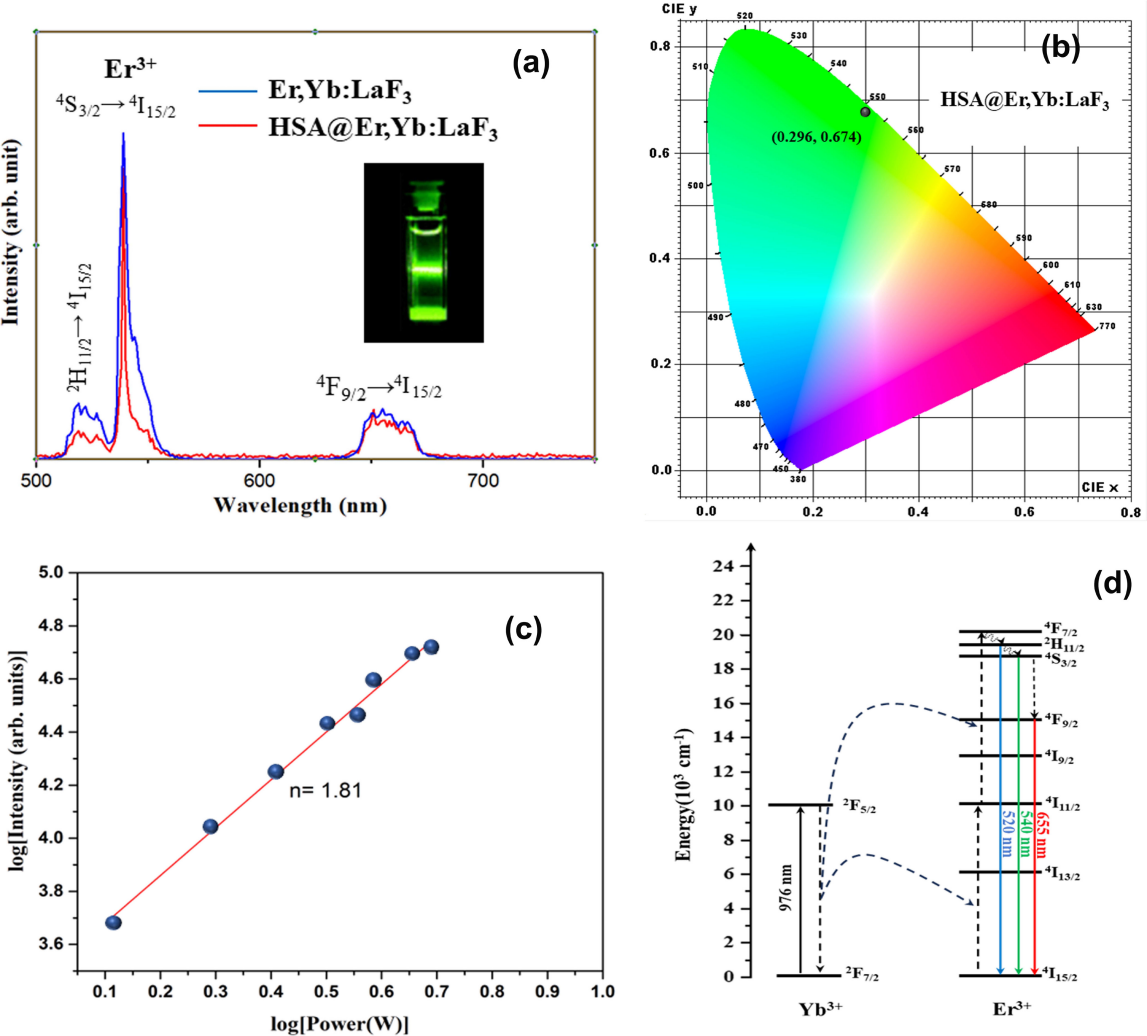
Optical characteristics: (a) PL emission spectra of bare and HSA‐coated Er,Yb:LaF_3_ NPs under 976 nm (1.5 W/cm^2^) excitation. (b) CIE chromaticity coordinate of HSA‐coated Er,Yb:LaF_3_ NPs. (c) Logarithmic dependence of UC emission intensity at 540 nm as a function of 976‐nm laser pump power for Er,Yb:LaF_3_ NPs. (d) Schematic of UC mechanism in Er,Yb:LaF_3_ NPs upon excitation with 976‐nm laser.

The PL emission spectra of Er,Yb:LaF_3_ samples, both bare and coated with HSA, were measured under excitation by a 976‐nm CW laser diode. As illustrated in Figure 6a, the UC emission spectra exhibit three distinct peaks centered at ~520, 540, and 655 nm. The peaks observed in the green region (~520 and 540 nm) correspond to the ^2^H_11/2_, ^4^S_3/2_ → ^4^I_15/2_ transitions, while the peak in the red region (~655 nm) originates from the ^4^F_9/2_ → ^4^I_15/2_ intrinsic transition of Er^3+^ ions [20, 41]. The spectral characteristics of both bare and HSA‐coated Er,Yb:LaF_3_ remain same (Figure 6a). This suggests that HSA functions as an ideal coating material, exhibiting minimal absorption of incident or emitted light.

Further, to correlate this spectrum with human visual perception of color, the Commission Internationale de l'Eclairage (CIE) chromaticity diagram is plotted in Figure 6b, along with the corresponding color coordinates (0.296, 0.674) of the emitted light.

### 3.5 Power‐Dependent UC Studies

To investigate the UC mechanism in Er,Yb:LaF_3_ NPs, the UC emission spectra were recorded as a function of excitation power, particularly focusing on the intense emission peak at 540 nm. The relationship between the UC emission intensity ($I_{\mathrm{UC}}$) and the incident laser power (P) can be described by the following equation [42]:

(1) $$I_{\mathrm{UC}}\;\alpha \;P^{n}$$

where *n* represents the number of photons required for the UC process.

As expected, an increase in UC emission intensity was observed with increasing pump power. To quantify this dependence, a double logarithmic plot (log–log plot) of emission intensity at 540 nm versus laser power is presented in Figure 6c. A linear fit to this data yielded a slope of 1.81. This calculated slope value, smaller than 2, may indicate cross‐relaxation and nonradiative losses in the NPs. These findings suggest that at least two NIR photons are involved in the UC process, resulting in the emission of a single visible photon (e.g., 540 nm).

### 3.6 Energy Transfer and UC Pathways

To further delve into the UC mechanism, Figure 6d presents a schematic energy level diagram of Yb^3+^ and Er^3+^ codoped LaF_3_, depicting the probable pathways for populating the excited states of Er^3+^.

Upon excitation with a 976‐nm CW laser, the NPs undergo ground state absorption (GSA) of Yb^3+^ ions (^2^F_7/2_ → ^4^F_5/2_) and Er^3+^ ions (^4^I_15/2_ → ^4^I_11/2_). Due to the higher absorption cross‐section of Yb^3+^, the excited ^4^I_11/2_ state of Er^3+^ is primarily populated by energy transfer from excited Yb^3+^ ions [26, 41, 43].

For populating ^4^F_7/2_ level of Er^3+^, two main pathways are possible:

- Excited state absorption (ESA): The ^4^I_11/2_ level absorbs another incoming 976‐nm NIR photon, promoting Er^3+^ to the ^4^F_7/2_ level.
- Energy transfer (ET): The excited ^2^F_5/2_ level of Yb^3+^ transfers its energy to the ^4^I_11/2_ level of Er^3+^, again leading to an increasing population of the ^4^F_7/2_ level.

However, the ^4^F_7/2_ level is short‐lived due to the presence of lower‐lying energy states [26]. Nonradiative transitions from ^4^F_7/2_ populate metastable ^2^H_11/2_ and ^4^S_3/2_ levels of Er^3+^ [26, 44]. Subsequent radiative transitions from these metastable levels to the ground state (^4^I_15/2_) result in the observed green emission at 520 and 540 nm, respectively.

For the red emission, the initial steps are similar, with GSA of Er^3+^ followed by population of the ^4^F_7/2_ level via ESA or ET [26, 41]. The crucial difference lies in the final relaxation pathway. Here, a combination of nonradiative transitions and radiative relaxation from a higher excited state (likely ^4^F_9/2_) leads to the characteristic red emission at 655 nm as illustrated in Figure 6a.

### 3.7 Cytotoxicity and Cellular Uptake Analysis

**FIGURE 7:**
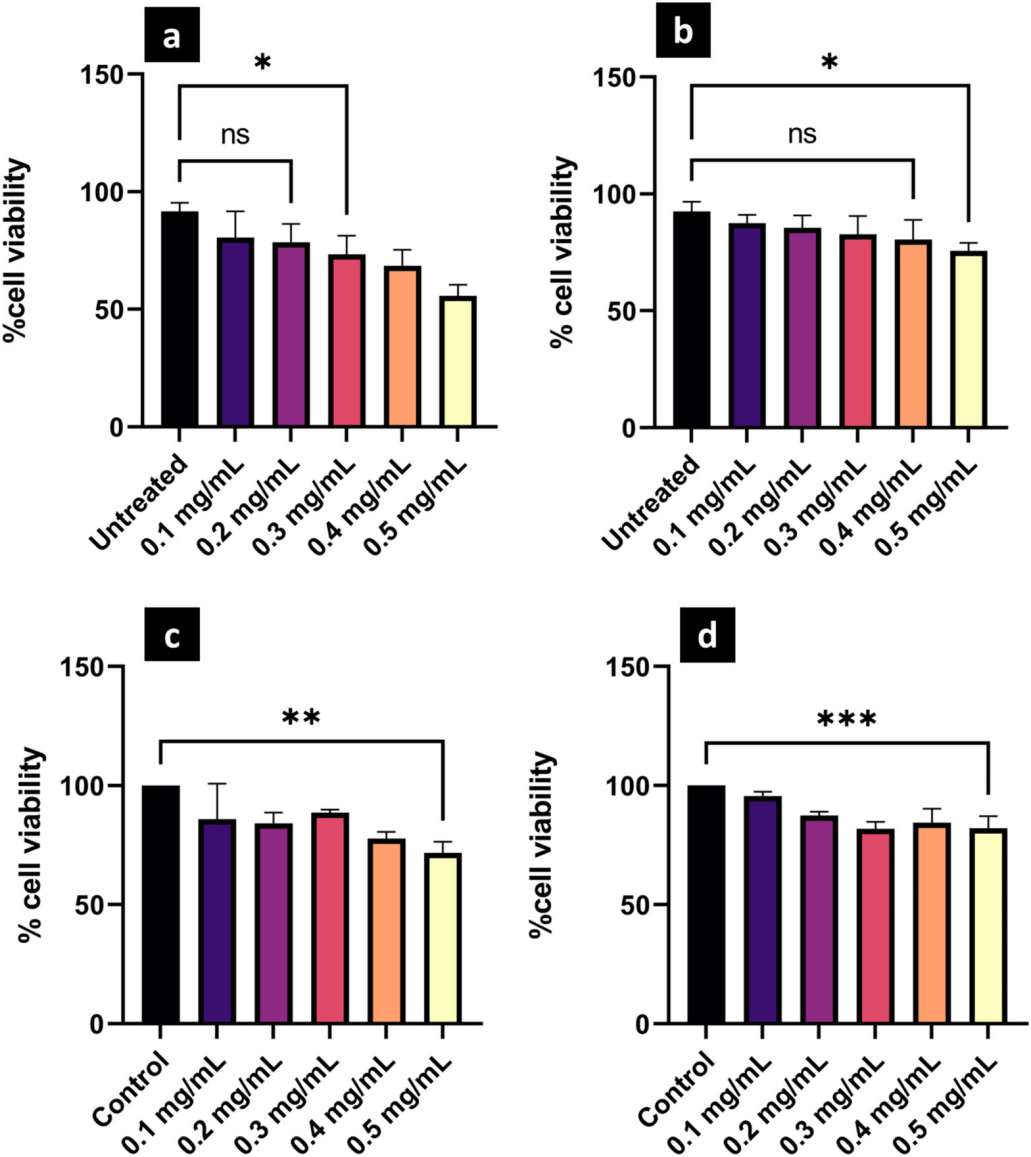
Cytotoxicity profile of (a) bare and (b) HSA‐coated Er,Yb:LaF_3_ incubated with HeLa cells and (c) bare and (d) HSA‐coated Er,Yb:LaF_3_ incubated with hADMS cells, all assessed after 24 h. *, **, and *** denote *p* < 0.05, *p* < 0.01, and *p* < 0.001, respectively.

Thorough investigation of cellular toxicity is essential, particularly for emerging NP classes where potential biological interactions remain largely unreported. Our investigation employed MTT assays to evaluate cell viability following 24‐h incubation of HeLa and Human Adipose‐Derived Mesenchymal Stem (hADMS) cells with bare and HSA‐coated Er,Yb:LaF_3_ NPs at 37°C. A range of UCNP concentrations was investigated, from a control group (0 mg/mL) to 0.5 mg/mL. Figure 7 shows a dose‐dependent response. In HeLa cells, viability remained high (> 80%) at concentrations between 0.1 and 0.4 mg/mL of HSA‐coated Er,Yb:LaF_3_. Compared to bare NPs, HSA‐coated NPs exhibit higher cell viability, indicating enhanced biocompatibility following HSA coating. Additionally, MTT assays performed on hADMSCs confirmed low cytotoxicity of HSA‐coated NPs up to 0.4 mg/mL, highlighting their safety for normal cells (one‐way ANOVA analysis, Figure 7). These findings suggest that HSA‐coated Er,Yb:LaF_3_ exhibits good biocompatibility at lower concentrations, but exposure to higher doses may induce cytotoxicity, emphasizing the need to optimize a suitable concentration range for NP usage.

The interaction of nanoparticles with cells is a complex and multifaceted process. Understanding these interactions is crucial for the development of bioimaging contrast agents. Thus, we investigated the cellular uptake of HSA‐coated Er,Yb:LaF_3_ by HeLa cervical cancer cells. Fluorescence spectroscopy, a powerful technique for quantifying intracellular nanoparticle concentration, was employed to determine the uptake dynamics.

**FIGURE 8:**
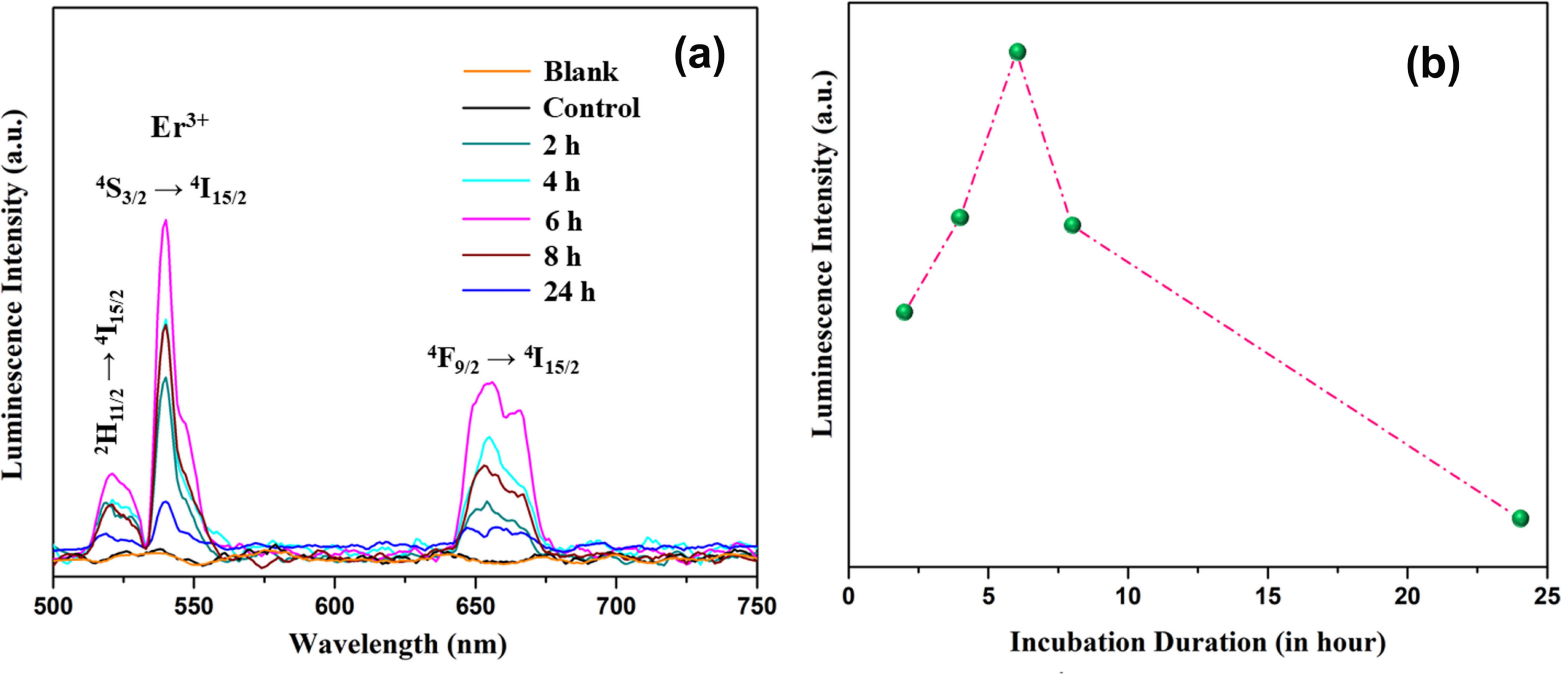
Cellular uptake study of HSA‐coated NPs: (a) PL spectra of HSA‐coated Er,Yb:LaF_3_ treated HeLa cell lysate under 976 nm (1.5 W/cm^2^) excitation. (b) Time‐dependent upconversion emission at 540 nm of HSA‐coated Er,Yb:LaF_3_ treated HeLa cells lysed suspension.

UCNPs are typically internalized by cells via endocytosis, where they bypass the cell membrane and enter the cytoplasm [45]. In this context, the intensity of UCNP fluorescence directly reflects their cellular concentration. As shown in Figure 8a, under 976‐nm excitation (1.5 W/cm^2^), the control sample (untreated cells) exhibits an emission profile similar to that of the blank (cell lysis solution), which ensures specific detection of UCNPs. The optical characteristics of the UC nanocomposite internalized within cells remained unchanged, as shown in Figure 8a. Upon excitation at 976 nm, GSA occurs in Yb^3+^ (^2^F_7/2_ → ^4^F_5/2_) and Er^3+^(^4^I_15/2_ → ^4^I_11/2_) ions. Subsequently, ET from Yb^3+^ and ESA in Er^3+^ facilitate the characteristic PL emissions of Er^3+^ at ~520, 540, and 655 nm as indicated in Figure 8a.

The observation revealed a time‐dependent uptake profile as shown in Figure 8b. Fluorescence intensity progressively increased, indicating a steady accumulation of HSA‐coated Er,Yb:LaF_3_ within the cells likely driven by endocytosis. This increase peaked at 6‐h posttreatment, signifying the maximum cellular uptake of UCNPs. Interestingly, following the peak, a decrease in fluorescence intensity was observed. This could be attributed to efflux (active expulsion) of UCNPs [46].

**FIGURE 9:**
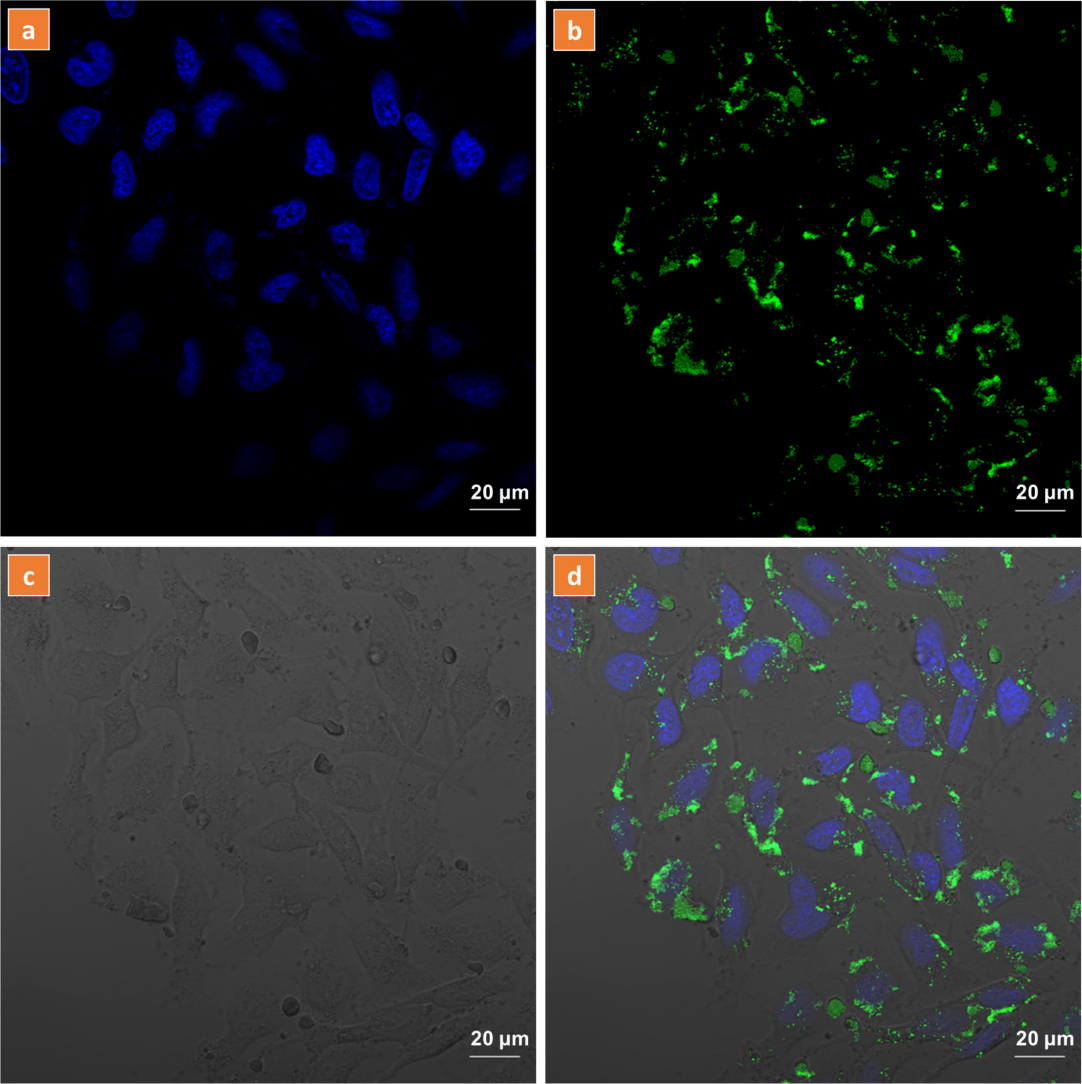
CLSM images of HeLa cells incubated with 0.3 mg/mL HSA‐coated Er,Yb:LaF_3_ nanocomposites for 6 h. Cells were counterstained with Hoechst nuclear dye and imaged using 405‐nm excitation for Hoechst and 488 nm for HSA@Er,Yb:LaF_3_. The scale bar represents 20 μm. (a) Hoechst nuclear staining channel, (b) HSA@Er,Yb:LaF_3_ channel, (c) transmitted light channel, and (d) merged image of all channels.

Further, the cellular uptake and visualization of HSA‐coated Er,Yb:LaF_3_ were evaluated using confocal laser scanning microscopy (CLSM). Figure 9 shows the CLSM image of HeLa cancer cells that were incubated with 0.3 mg/mL of UCNPs for 6 h. Due to the unavailability of the 976‐nm source in the existing setup, HeLa cells were excited with a 488‐nm wavelength corresponding to the Er^3+^ (^4^I_15/2_ → ^4^F_7/2_) excitation using an Ar laser (PL emission spectra under 488 nm are included in Figure S1). The characteristic luminescence emission in the green spectrum (530–560 nm) was captured by photomultiplier tube (PMT) detectors. To visualize the nuclei, the cells were prestained with Hoechst 33342 dye and excited with a 405 nm laser diode, resulting in a blue emission that is evident in the acquired images. The CLSM image clearly shows cytoplasmic uptake of the nanophosphors.

Overall, this study demonstrates that HSA‐coated Er,Yb:LaF_3_ UCNPs are taken up by HeLa cells, making them potentially valuable tools for cellular imaging applications.

## 4 Conclusions

In this study, 2 mol% Er^3+^ and 20 mol% Yb^3+^ codoped LaF_3_ NPs were successfully synthesized using a hydrothermal method. HSA coating successfully improved the biocompatibility of UCNPs for bioimaging applications, while preserving their favorable optical properties. The synthesized UCNPs exhibited good UC luminescence properties, with emissions in the green and red regions upon NIR (976 nm) excitation. Investigations into the UC mechanism suggest a dependence on at least two NIR photons for single visible photon emission. Cytotoxicity studies demonstrated good biocompatibility of the UCNPs at lower concentrations with HeLa cells. Furthermore, cellular uptake experiments revealed a time‐dependent accumulation of UCNPs within the cells, indicating their successful internalization. These findings highlight the promise of these engineered UCNPs for bioimaging applications. Their efficient UC properties, combined with good biocompatibility and cellular uptake, suggest their potential as contrast agents for deeper tissue penetration and minimized phototoxicity compared to traditional methods. Further research is warranted to optimize their performance and explore their *in vivo* imaging capabilities.

## Conflicts of Interest

The authors declare no conflicts of interest.

## Supporting information


**Figure S1:** PL spectra of HSA coated LaF_3_: 2%Er, 20%Yb nanophosphor in DI water and loaded in HeLa lysed suspension under 488‐nm excitation.

## Data Availability

The data that support the findings of this study are available on request from the corresponding author. The data are not publicly available due to privacy or ethical restrictions.

## References

[1] J. C. Hsu , Z. Tang , O. E. Eremina , et al., “Nanomaterial‐Based Contrast Agents,” Nature Reviews Methods Primers 3 (2023): 30, 10.1038/s43586-023-00211-4. PMC10732545 38130699

[2] T. Beyer , L. Bidaut , J. Dickson , et al., “What Scans We Will Read: Imaging Instrumentation Trends in Clinical Oncology,” Cancer Imaging 20 (2020): 38, 10.1186/s40644-020-00312-3. 32517801 PMC7285725

[3] B. Sun , X. Zhen , and X. Jiang , “Development of Mesoporous Silica‐Based Nanoprobes for Optical Bioimaging Applications,” Biomaterials Science 9 (2021): 3603–3620, 10.1039/D1BM00204J. 34008597

[4] G. M. Saladino , C. Vogt , Y. Li , et al., “Optical and X‐Ray Fluorescent Nanoparticles for Dual Mode Bioimaging,” ACS Nano 15 (2021): 5077–5085, 10.1021/acsnano.0c10127. 33587608 PMC8028327

[5] P. Sowmiya , T. S. Dhas , D. Inbakandan , et al., “Optically Active Organic and Inorganic Nanomaterials for Biological Imaging Applications: A Review,” Micron 172 (2023): 103486, 10.1016/j.micron.2023.103486. 37262930

[6] E. M. Mettenbrink , W. Yang , and S. Wilhelm , “Bioimaging With Upconversion Nanoparticles,” Advanced Photonics Research 3 (2022): 2200098, 10.1002/adpr.202200098. 36686152 PMC9858112

[7] B. Li , A. A. Ansari , A. K. Parchur , and R. Lv , “Exploring the Influence of Polymeric and Non‐Polymeric Materials in Synthesis and Functionalization of Luminescent Lanthanide Nanomaterials,” Coordination Chemistry Reviews 514 (2024): 215922, 10.1016/j.ccr.2024.215922.

[8] R. Lv , Y. Wang , B. Lin , et al., “Targeted Luminescent Probes for Precise Upconversion/NIR II Luminescence Diagnosis of Lung Adenocarcinoma,” Analytical Chemistry 93 (2021): 4984–4992, 10.1021/acs.analchem.1c00374. 33705098

[9] A. A. Ansari , A. K. Parchur , Y. Li , et al., “Cytotoxicity and Genotoxicity Evaluation of Chemically Synthesized and Functionalized Upconversion Nanoparticles,” Coordination Chemistry Reviews 504 (2024): 215672, 10.1016/j.ccr.2024.215672.

[10] H. Dong , L.‐D. Sun , and C.‐H. Yan , “Basic Understanding of the Lanthanide Related Upconversion Emissions,” Nanoscale 5 (2013): 5703–5714, 10.1039/C3NR34069D. 23423120

[11] B. del Rosal , I. Villa , D. Jaque , and F. Sanz‐Rodríguez , “In Vivo Autofluorescence in the Biological Windows: The Role of Pigmentation,” Journal of Biophotonics 9 (2016): 1059–1067, 10.1002/jbio.201500271. 26576035

[12] Kenry , Y. Duan , and B. Liu , “Recent Advances of Optical Imaging in the Second Near‐Infrared Window,” Advanced Materials 30 (2018): 1802394, 10.1002/adma.201802394. 30182451

[13] M. Bettinelli , L. Carlos , and X. Liu , “Lanthanide‐Doped Upconversion Nanoparticles,” Physics Today 68 (2015): 38–44, 10.1063/PT.3.2913.

[14] P. Deshmukh , S. Satapathy , M. K. Singh , Y. P. Kumar , and P. K. Gupta , “Tb3+/Yb3+ Co‐Doped Y2O3 Upconversion Transparent Ceramics: Fabrication and Characterization for IR Excited Green Emission,” Journal of the European Ceramic Society 37 (2017): 239–242, 10.1016/j.jeurceramsoc.2016.07.031.

[15] W. D. A. M. de Boer , D. Timmerman , K. Dohnalová , et al., “Red Spectral Shift and Enhanced Quantum Efficiency in Phonon‐Free Photoluminescence From Silicon Nanocrystals,” Nature Nanotechnology 5 (2010): 878–884, 10.1038/nnano.2010.236. 21113157

[16] B. Chen and F. Wang , “Recent Advances in the Synthesis and Application of Yb‐Based Fluoride Upconversion Nanoparticles,” Inorganic Chemistry Frontiers 7 (2020): 1067–1081, 10.1039/C9QI01358J.

[17] Y. Li , Y. Zhang , G. Hong , and Y. Yu , “Upconversion Luminescence of Y2O3:Er3+, Yb3+ Nanoparticles Prepared by a Homogeneous Precipitation Method,” Journal of Rare Earths 26 (2008): 450–454, 10.1016/S1002-0721(08)60116-7.

[18] T. K. Anh , V. T. T. Ha , N. T. Huong , et al., “Synthesis and Characterizations of Upconverting Luminescent Er3+/Yb3+: Gd2O3 Uniform Nanospheres for Biomedical Applications,” Physica Scripta 99 (2024): 1059d5, 10.1088/1402-4896/ad7c03.

[19] I. Kamińska , A. Wosztyl , P. Kowalik , et al., “Synthesis and Characterization of Gd2O3: Er3+, Yb3+ Doped With Mg2+, Li+ Ions—Effect on the Photoluminescence and Biological Applications,” Nanotechnology 32 (2021): 245705, 10.1088/1361-6528/abed02. 33690193

[20] X. Cheng , Y. Pan , Z. Yuan , et al., “Er3+ Sensitized Photon Upconversion Nanocrystals,” Advanced Functional Materials 28 (2018): 1800208, 10.1002/adfm.201800208.

[21] P. Deshmukh , S. Satapathy , M. K. Singh , M. P. Kamath , and A. K. Karnal , “Effect of Er and Dy on IR‐Visible Up‐Conversion Luminescence Properties of (Er0.01Dy0.01La0.01Zr0.02Y0.95)2O3 Transparent Ceramic,” Ceramics International 43 (2017): 14257–14262, 10.1016/j.ceramint.2017.07.174.

[22] B. Chen and F. Wang , “Combating Concentration Quenching in Upconversion Nanoparticles,” Accounts of Chemical Research 53 (2020): 358–367, 10.1021/acs.accounts.9b00453. 31633900

[23] S. Dubey , P. Deshmukh , S. Satapathy , M. K. Singh , and P. K. Gupta , “Effect of Mg Doping in Sr2SiO4:Eu2+ Nanophosphors for Blue and White Emission at Near‐UV Excitation,” Luminescence 32 (2017): 839–844, 10.1002/bio.3260. 28067013

[24] L. M. Wiesholler , F. Frenzel , B. Grauel , C. Würth , U. Resch‐Genger , and T. Hirsch , “Yb,Nd,Er‐Doped Upconversion Nanoparticles: 980 nm Versus 808 nm Excitation,” Nanoscale 11 (2019): 13440–13449, 10.1039/C9NR03127H. 31287476

[25] S. T. Dibaba , X. Ge , W. Ren , and L. Sun , “Recent Progress of Energy Transfer and Luminescence Intensity Boosting Mechanism in Nd^3+^−Sensitized Upconversion Nanoparticles,” Journal of Rare Earths 37 (2019): 791–805, 10.1016/j.jre.2019.02.001.

[26] F. T. Rabouw , P. T. Prins , P. Villanueva‐Delgado , M. Castelijns , R. G. Geitenbeek , and A. Meijerink , “Quenching Pathways in NaYF_4_:Er^3+^,Yb^3+^ Upconversion Nanocrystals,” ACS Nano 12 (2018): 4812–4823, 10.1021/acsnano.8b01545. 29648802 PMC5968434

[27] M. Pokhrel , S. K. Gupta , A. Perez , et al., “Up‐ and Down‐Convertible LaF_3_:Yb,Er Nanocrystals With a Broad Emission Window From 350 nm to 2.8 μm: Implications for Lighting Applications,” ACS Applied Nano Materials 4 (2021): 13562–13572, 10.1021/acsanm.1c03023.

[28] V. Tamilmani , A. K. Soni , V. K. Rai , B. U. Nair , and K. J. Sreeram , “NIR‐Excited Imaging of Cervical Cancer Cells Using Biocompatible LaF_3_:Er^3+^,Yb^3+^ Upconversion Nanophosphors,” Journal of Chemical Sciences 129 (2017): 1929–1940, 10.1007/s12039-017-1401-4.

[29] D. Sleep , J. Cameron , and L. R. Evans , “Albumin as a Versatile Platform for Drug Half‐Life Extension,” Biochimica et Biophysica Acta (BBA) ‐ General Subjects 1830 (2013): 5526–5534, 10.1016/j.bbagen.2013.04.023. 23639804

[30] T. Peters, Jr. , All About Albumin: Biochemistry, Genetics, and Medical Applications (Academic press, 1995).

[31] P. Deshmukh , R. K. Deo , A. Ahlawat , et al., “Spectroscopic Investigation of Upconversion and Downshifting Properties LaF3:Tb3+,Yb3+: A Dual Mode Green Emitter Nanophosphor,” Journal of Alloys and Compounds 859 (2021): 157857, 10.1016/j.jallcom.2020.157857.

[32] M. S. Maleki , O. Moradi , and S. Tahmasebi , “Adsorption of Albumin by Gold Nanoparticles: Equilibrium and Thermodynamics Studies,” Arabian Journal of Chemistry 10 (2017): S491–S502, 10.1016/j.arabjc.2012.10.009.

[33] A. Zalkin and D. H. Templeton , “Refinement of the Trigonal Crystal Structure of Lanthanum Trifluoride With Neutron Diffraction Data,” Acta Crystallographica Section B 41 (1985): 91–93, 10.1107/S0108768185001689.

[34] R. D. Shannon , “Revised Effective Ionic Radii and Systematic Studies of Interatomic Distances in Halides and Chalcogenides,” Acta Crystallographica Section A 32 (1976): 751–767, 10.1107/S0567739476001551.

[35] N. Abdollahpour , V. Soheili , M. R. Saberi , and J. Chamani , “Investigation of the Interaction Between Human Serum Albumin and Two Drugs as Binary and Ternary Systems,” Eur J Drug Metab Pharmacokinet 41 (2016): 705–721, 10.1007/s13318-015-0297-y. 26328807

[36] M. Beg , A. Maji , A. K. Mandal , et al., “Green Synthesis of Silver Nanoparticles Using *Pongamia pinnata* Seed: Characterization, Antibacterial Property, and Spectroscopic Investigation of Interaction With Human Serum Albumin,” Journal of Molecular Recognition 30 (2017): e2565, 10.1002/jmr.2565. 27677774

[37] S. Ji , T. Jiang , K. Xu , and S. Li , “FTIR Study of the Adsorption of Water on Ultradispersed Diamond Powder Surface,” Applied Surface Science 133 (1998): 231–238, 10.1016/S0169-4332(98)00209-8.

[38] F. Tavakkoli , M. Zahedifar , and E. Sadeghi , “Effect of LaF3: Ag Fluorescent Nanoparticles on Photodynamic Efficiency and Cytotoxicity of Protoporphyrin IX Photosensitizer,” Photodiagnosis and Photodynamic Therapy 21 (2018): 306–311, 10.1016/j.pdpdt.2018.01.009. 29331661

[39] I. Yousuf , M. Bashir , F. Arjmand , and S. Tabassum , “Multispectroscopic Insight, Morphological Analysis and Molecular Docking Studies of CuII‐Based Chemotherapeutic Drug Entity With Human Serum Albumin (HSA) and Bovine Serum Albumin (BSA),” Journal of Biomolecular Structure & Dynamics 37 (2019): 3290–3304, 10.1080/07391102.2018.1512899. 30124142

[40] H. Alhazmi , “FT‐IR Spectroscopy for the Identification of Binding Sites and Measurements of the Binding Interactions of Important Metal Ions With Bovine Serum Albumin,” Scientia Pharmaceutica 87 (2019): 5, 10.3390/scipharm87010005.

[41] B. P. Kore , A. Kumar , L. Erasmus , et al., “Energy Transfer Mechanisms and Optical Thermometry of BaMgF4:Yb3+,Er3+ Phosphor,” Inorganic Chemistry 57 (2018): 288–299, 10.1021/acs.inorgchem.7b02436. 29227098

[42] J. F. Suyver , A. Aebischer , S. Garcia‐Revilla , P. Gerner , and H. U. Güdel , “Anomalous Power Dependence of Sensitized Upconversion Luminescence,” Physical Review B: Condensed Matter 71 (2005): 125123, https://api.semanticscholar.org/CorpusID:121549990.

[43] J. Liu , H. Deng , F. Lv , et al., “Up−/Downconversion Luminescence in Gd2O3:Yb3+/Er3+ Nanocrystals: Emission Manipulation and Energy Transfer Phenomena,” Journal of Luminescence 206 (2019): 486–491, 10.1016/j.jlumin.2018.10.074.

[44] C. Renero‐Lecuna , R. Martín‐Rodríguez , R. Valiente , et al., “Origin of the High Upconversion Green Luminescence Efficiency in β‐NaYF4:2%Er3+,20%Yb3+,” Chemistry of Materials 23 (2011): 3442–3448, 10.1021/cm2004227.

[45] D. Chávez‐García , K. Juárez‐Moreno , C. H. Campos , J. B. Alderete , and G. A. Hirata , “Upconversion Rare Earth Nanoparticles Functionalized With Folic Acid for Bioimaging of MCF‐7 Breast Cancer Cells,” Journal of Materials Research 33 (2018): 191–200, 10.1557/jmr.2017.463.

[46] E. Voronovic , A. Skripka , G. Jarockyte , et al., “Uptake of Upconverting Nanoparticles by Breast Cancer Cells: Surface Coating Versus the Protein Corona,” ACS Appl Mater Interfaces 13 (2021): 39076–39087, 10.1021/acsami.1c10618. 34378375 PMC8824430

