# Supplementary figures and images for: NIR‐Excited Imaging of Cervical Cancer Cells Using Biocompatible LaF3:Er3+,Yb3+ Upconversion Nanophosphors
Source: Luminescence. 2025 Jul 25;40(7):e70269. doi: 10.1002/bio.70269 (PMC12290501; doi:10.1002/bio.70269)

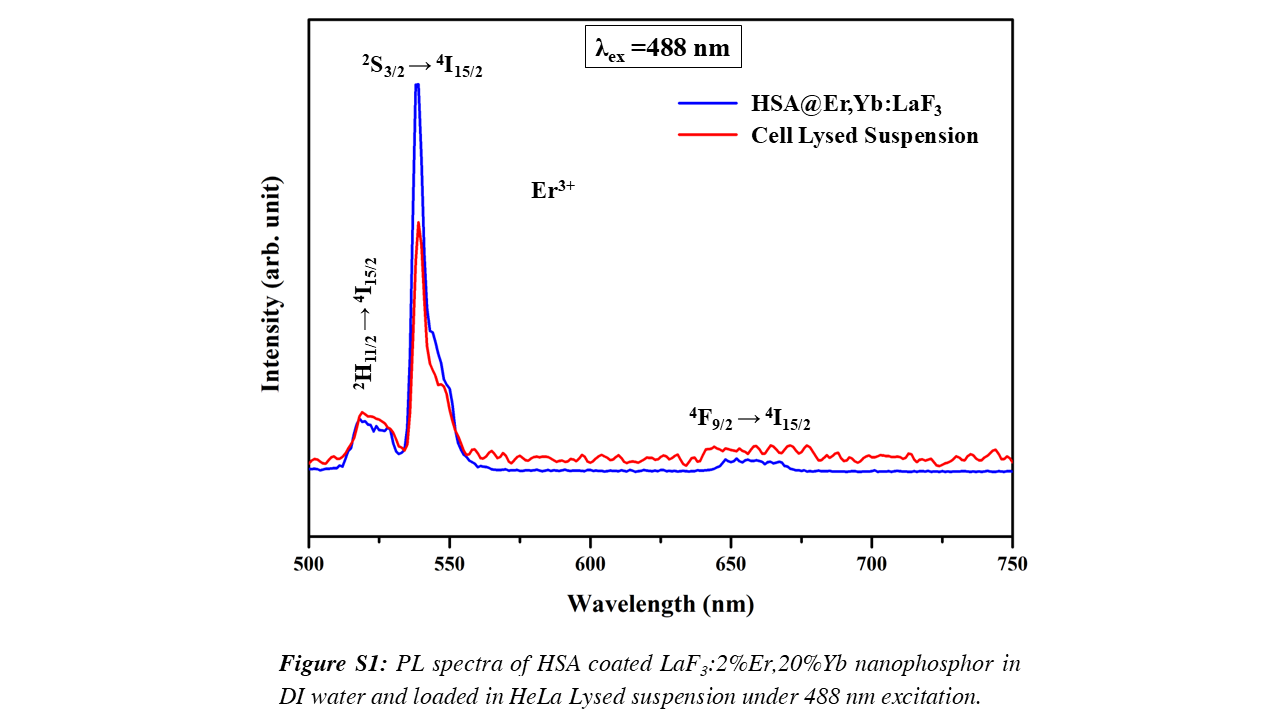

Supplement: Supplementary file 1 — Figure S1: PL spectra of HSA coated LaF3: 2%Er, 20%Yb nanophosphor in DI water and loaded in HeLa lysed suspension under 488‐nm excitation. [file BIO-40-e70269-s001.png]
